# The Georgia State Commission on Tuberculosis

**Published:** 1905-02

**Authors:** 


### THE GEORGIA STATE COMMISSION ON TUBERCULOSIS.

THE work of distributing blanks for the purpose of collecting statistics on tuberculosis in the State has been going on for more than a month, and so far as their names can be got at through the several sources of information every physician in the State has received one. The returns have been exceedingly meager. In some instances the blank has been mailed with stamped envelope for reply, but more frequently than not both stamp and blank have disappeared.

The Commission's work is a serious matter—one not to be lightly put aside. It seeks to ascertain the status of tuberculosis in the State and to recommend means to suppress it. This information must come from the doctors, and from no other source. The success of the Commission's labors depends solely upon them. It is obvious that a report which is not based upon a tangible statistical support is emasculate, ineffective, and practically worthless. Therefore every reader of this journal in Georgia is earnestly requested to send in his report to the district member of the Commission or to the editor of the JOURNAL-RECORD without delay. If he has not received one of the blanks, it will be furnished him promptly on application. Do not put the matter off, but attend to it now.

